# Does Angiotensin II Peak in Response to SARS-CoV-2?

**DOI:** 10.3389/fimmu.2020.577875

**Published:** 2021-01-14

**Authors:** Léder Leal Xavier, Paula Fernanda Ribas Neves, Lisiê Valeria Paz, Laura Tartari Neves, Pamela Brambilla Bagatini, Luís Fernando Saraiva Macedo Timmers, Alberto Antônio Rasia-Filho, Régis Gemerasca Mestriner, Andrea Wieck

**Affiliations:** ^1^ Laboratório de Biologia Celular e Tecidual, Programa de Pós-Graduação em Biologia Celular e Molecular, Escola de Ciências da Saúde e da Vida, Pontifícia Universidade Católica do Rio Grande do Sul, PUCRS, Porto Alegre, Brazil; ^2^ Programa de Pós-Graduação em Biotecnologia (PPGBiotec), Programa de Pós-Graduação em Ciências Médicas (PPGCM), Universidade do Vale do Taquari-UNIVATES, Lajeado, Brazil; ^3^ Departamento de Ciências Básicas da Saúde/Fisiologia, Universidade Federal de Ciências da Saúde de Porto Alegre-UFCSPA, Porto Alegre, Brazil

**Keywords:** COVID-19, SARS-CoV-2, Angiotensin-converting enzyme 2, angiotensin-II, immune activation, immune response

## Abstract

Human infection by the SARS-CoV-2 is causing the current COVID-19 pandemic. With the growing numbers of cases and deaths, there is an urgent need to explore pathophysiological hypotheses in an attempt to better understand the factors determining the course of the disease. Here, we hypothesize that COVID-19 severity and its symptoms could be related to transmembrane and soluble Angiotensin-converting enzyme 2 (tACE2 and sACE2); Angiotensin II (ANG II); Angiotensin 1-7 (ANG 1-7) and angiotensin receptor 1 (AT1R) activation levels. Additionally, we hypothesize that an early peak in ANG II and ADAM-17 might represent a physiological attempt to reduce viral infection *via* tACE2. This viewpoint presents: (1) a brief introduction regarding the renin-angiotensin-aldosterone system (RAAS), detailing its receptors, molecular synthesis, and degradation routes; (2) a description of the proposed early changes in the RAAS in response to SARS-CoV-2 infection, including biological scenarios for the best and worst prognoses; and (3) the physiological pathways and reasoning for changes in the RAAS following SARS-CoV-2 infection.

## Introduction

The current COVID-19 pandemic, caused by SARS-CoV-2 infection, has affected virtually all the countries in the world (1). With new cases and deaths being reported daily, there is an urgent need to understand the pathophysiological basis of the disease’s progression, especially in the most severe cases. This may accelerate the discovery of effective treatments, thus increasing survival rates and consequently alleviating the disease’s social impact. There is considerable biochemical, physiological and pathological evidence to show that the renin-angiotensin-aldosterone system (RAAS) plays a pivotal role in COVID-19. Here, we propose two hypotheses regarding the RAAS, its related receptors, enzymatic synthesis and degradation routes to address Angiotensin II (ANG II) involvement in the range of clinical prognoses of COVID-19.

This study is divided into three main parts. The first provides a description of the RAAS, detailing its receptors, molecular synthesis and degradation routes. The second deals with changes in the RAAS during SARS-CoV-2 infection, including biological scenarios for the best and worst COVID-19 prognoses. The final section discusses the physiological pathways and reasoning for the proposed early changes in the RAAS in response to SARS-CoV-2 infection and COVID-19 development.

### The RAAS

The RAAS regulates blood composition and circulating volume, having a role in blood pressure homeostasis in various animal species (2). The classic RAAS pathway involves the production of Renin by juxtaglomerular cells whose only substrate is angiotensinogen, forming Angiotensin I (ANG I) (3). In turn, ANG I is then cleaved into ANG II by Angiotensin-Converting Enzyme (ACE), which is primarily transcribed as a membrane-bound enzyme and later detached from the cell membrane by proteolytic cleavage (4, 5). Specifically, the C-terminal portion of ACE (also known as ACE/Kininase II) is responsible for ANG I metabolization to ANG II, while the N-terminal portion cleaves Bradykinin into other peptides (6, 7). However, ANG II might also be generated by other enzymes in tissue-specific RAAS (cardiovascular, pulmonary and renal) (8). The main enzyme involved in ANG II generation in tissue-specific RAAS are serine proteases (specially kallikrein-like enzymes also called tonins), and also cathepsin G and chymase (9). In the kidneys, 40% of ANG II is generated by non-ACE pathways, while in human heart, coronary arteries and atherosclerotic aorta, chymase is the dominant generator of ANG II, suggesting that these non-classical pathways might have a role in the development of several diseases (9, 10). The best described physiological effects of ANG II involves its binding to AT1 receptors (AT1R), leading to vasoconstriction and reducing renal sodium excretion, *via* aldosterone stimulation (11).

A recent description of the RAAS has revealed several ANG II derived peptides, highlighting the importance of this system as well as providing a better understanding of its role (3). ACE2, which was initially described as an ACE homologous, sharing 40% sequence similarity with ACE (12), counteracts the effects of ANG II by converting ANG I into Angiotensin 1-9 (ANG 1-9), and ANG II into Angiotensin 1-7 (ANG 1-7) (13). While binding to both ANG I and II, ACE2 preferentially binds to ANG II, with a catalytic efficiency 400 times greater than for ANG I. Consequently, plasma levels of ANG 1-7 are higher than those of the other isoforms, such as ANG 1-9 (12, 14). ANG 1-7, by the actions of ACE and aminopeptidases, generate two other peptides, Angiotensin 1-5 and Angiotensin 2-7, respectively (3). Angiotensin 2-7 will then generate Angiotensin 3-7 *via* aminopeptidases action, while Angiotensin 1-5 is converted into Angiotensin 1-4 by neprilysin or carboxipeptidases. Finally, both Angiotensins 3-7 and 1-4 are converted into Angiotensin 3-4 by aminopeptidases and endopeptidases (3). Besides ANG 1-7 generation, ANG II, when cleaved by a carboxylase, will generate Angiotensin A, whereas when cleaved by aminopeptidase A, ANG II generates Angiotensin III (3). Angiotensin III is then cleaved by aminopeptidase N, forming Angiotensin IV, which is then cleaved by another aminopeptidase, forming angiotensin 5-8. The pathway ends with angiotensin 5-8 being processed by a carboxypeptidase, generating angiotensin 5-7 (3). Most of these new RAAS members are ANG II-derived peptides, and also exert their actions *via* AT1R and AT2R, having local physiological effects that can be similar to those produced by ANG II, according to the tissues and systems were they occur.

**Figure 1:**
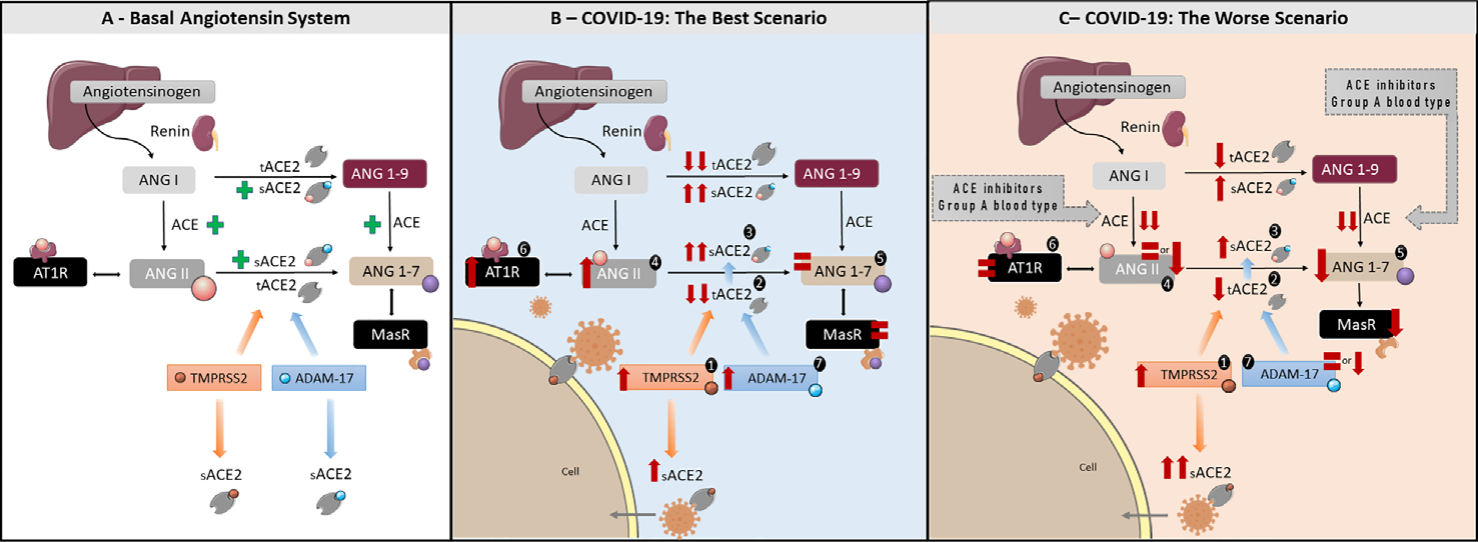
The roles of the RAAS in health and COVID-19: A better and worse scenarios—**(A)** Basal angiotensin system. Figure representing the classic RAAS pathway. Angiotensin I (ANG I) is produced from angiotensinogen by renin. ANG I is converted to Angiotensin II (ANG II) by Angiotensin-Converting Enzyme (ACE). ANG II exerts its actions by binding to AT1R. Angiotensin-Converting Enzyme 2 (ACE2), is responsible for converting ANG I to Angiotensin 1-9 (ANG 1-9) and ANG II to Angiotensin 1-7 (ANG 1-7). ANG 1-7 has opposite effects to ANG II, counterbalancing ANG II physiological outcome. ADAM 17 and TPMRSS2 are sheddases responsible for cleaving tACE2 into its soluble form, sACE2. ADAM17 cleaves tACE2 in a constitutively manner, while TMPRSS2 is implicated in SARS-CoV-2 infection, supporting virus entry the cell host. **(B)** COVID-19 Best Scenario (for a better understanding, follow the order of numbers). Increased TMPRSS2 activity due to viral entry resulting in reduced tACE2 and consequent increase in sACE2, unable to convert ANG II in ANG 1-7. This will lead to increased ANG II levels, and increased AT1R activation, resulting in higher ADAM-17 activity. Higher ADAM-17 activity will increase biologically active sACE2 levels, able to convert ANG II in ANG 1-7, resulting in no alteration in ANG 1-7 levels. In this scenario, the intense tACE2 cleavage promoted by ANG II/AT1R/ADAM-17 axis will reduce virus entry into the cells. **(C)** COVID-19 Worst Scenario Note that this scenario is closely related to an incapacity in promote the ANG II peak or AT1R down-regulation, as suggested to the use of ACE inhibitors, genetic factors (i.e. A blood type), diabetes mellitus and cardiovascular diseases. (for a better understanding, follow the order of numbers). Increased TMPRSS2 activity due to viral entry resulting in a mild reduction tACE2 as well as a mild increase in sACE2, unable to convert ANG II into ANG 1-7. Consequently, ANG II levels will remain equal or even decrease, as well as AT1R activation, and resulting in ANG 1-7 decrease. In this scenario, the equal or low levels of ANG II, AT1R, and ADAM-17 activities will be associated to a mild decrease in tACE2 and a substantial increase in sACE2 produced by TPMRSS2, increasing the virus entry into the cells.

ACE2 is expressed in multiple human epithelial cell types, including vascular endothelial cells, nasal and intestinal epithelial cells, and within the airways, in alveolar epithelial type II cells of the lung parenchyma (15). It is a metalloproteinase type I transmembrane enzyme, characterized by a small cytoplasmic domain, a transmembrane region and an ectodomain (16). Due to conformational differences in the catalytic site, ACE2 is not blocked by classic ACE inhibitors (12) nor angiotensin-receptor blockers. The ACE2 ectodomain is recognized as a cleavage site for ADAM-17 and TMPRSS2 sheddases (17). ADAM-17 is a disintegrin and metalloproteinase 17 domain (17) that cleaves ectodomains of diverse cytokines, receptors and enzymes anchored in cell membranes (18). TMPRSS2 is a type II serine protease, acting in conjunction with ADAM-17. Both enzymes share the same specificity for transmembrane ACE2 (tACE2), however, the final product, soluble ACE2 (sACE2), is different (19). ADAM-17 cleavage leads to the shedding of a form of sACE2 that is still biologically active, being able to cleave ANG II and counteracting its effects, but also blocking circulating viral particles (14). Studies using recombinant sACE2 have demonstrated that it also cleaves ANG I into ANG 1-9, which also counteracts ANG II effects. However, little is known about physiologically produced sACE2 and ANG I cleavage (20). tACE2 cleavage by TMPRSS2, on the other hand, does not release the biologically active form of the enzyme (21) and has been shown to be involved in SARS-CoV and SARS-CoV-2 infections, being the main pathway by which the virus enters the cell (13, 22–24). Therefore, the balance between the two sACE2 forms generated by both enzymes might have an important role in COVID-19 outcomes. This is summarized in **Figure 1A** and is relevant to COVID-19 symptoms.

## Putative Changes in the Raas Related TO COVID-19

The putative pathways involving the RAAS and disturbances caused by SARS-CoV-2 infection are summarized in **Figures 1B, C**. **Figure 1B** summarizes our hypothesis regarding the best prognosis in COVID-19, while **Figure 1C** details the worst prognosis. The best prognosis involves an early ANG II peak in response to SARS-CoV-2 infection, being related to outcomes were the inflammatory process is controlled, with little tissue damage, no or short hospitalization as well as mild or asymptomatic COVID-19. The worst prognosis would result from the inability of the organism to produce an ANG II peak as soon as SARS-CoV-2 infects the cell. It is related to those cases where severe COVID-19 develops, with the presence of a cytokine storm, tissue damage, organ failure and long hospitalization, frequently culminating in death. As highlighted, the key point is a peak in ANG II following SARS-CoV-2 infection and/or a strong AT1R activation. A step-by-step description of the pathways that may lead to a best prognosis is described in **Figure 1B**, below. The hypotheses outlined here will be supported by data from the literature and discussed in more depth in the discussion section.

**Figure 1B** sequence:

1. Increase in TMPRSS2 activity. Origin: TMPRSS2 activation by Sars-CoV-2 (25–27).
2. Strong decrease in tACE2 levels. Origins: a) SARS-CoV-2 internalization, together with tACE2 (28); b) tACE2 shedding *via* TMPRSS2 cleavage induced by SARS-CoV-2 (13, 22, 24); c) tACE2 shedding *via* ADAM-17 cleavage (13, 16, 22, 24) induced after AT1R activation by ANG II (11).
3. Strong increases in sACE2 levels. Origin: a) tACE2 shedding *via* TMPRSS2 cleavage induced by SARS-CoV-2 (13, 22, 24); b) tACE2 shedding *via* ADAM-17 cleavage induced after AT1R activation by ANG II (11).
4. Increase in ANG II levels. Origins: a) Decrease in tACE2 levels and b) A possible partial blockage of sACE2 catalytic activity by SARS-CoV-2 infection. Both factors reduce ANG II metabolization to ANG 1-7 (29) (see # 2).
5. ANG 1-7 levels remain equal. Origin: a) Decrease in tACE2 levels and b) Increase of biologically active sACE2 levels originated from ADAM-17 cleavage (14, 21) (see # 2 and #4).
6. Increased AT1R activation. Origin: Increase in ANG II levels (11) (see #4).
7. Increase in ADAM-17 activity. Origin: Increase in ANG II levels and, consequently, AT1R activation (11) (see # 4 and 6).

On the other hand, an absent/low ANG II peak, or absent AT1R activation and, consequent low ADAM-17 activation would lead to a worst condition associated with COVID-19. In this situation, sACE2 levels resultant from TMPRSS2 would be higher than its biologically active form, originated from ADAM-17 cleavage, thus facilitating SARS-CoV-2 infection (**Figure 1C**).

**Figure 1C** sequence:

1. Increase in TMPRSS2 activity. Origin: TMPRSS2 activation by SARS-CoV-2 (25–27).
  Observation: Note that, in this case, the higher tACE2 levels present when compared to the better prognosis, allows more TMPRSS2 activation due to SARS-CoV-2 binding and an increase in virus entry (21).
2. Mild decrease in tACE2 levels. Origins: a) SARS-CoV-2 internalization, together with tACE2 (28); b) tACE2 shedding *via* TMPRSS2 cleavage induced by SARS-CoV-2 (25–27). Observation: Note that, in this case, tACE2 shedding *via* ADAM-17 cleavage, induced by ANG II, is reduced when compared to the better choice model.
3. Mild increase in sACE2 levels. Origin: a) tACE2 shedding *via* TMPRSS2 cleavage induced by SARS-CoV-2 (25–27). Observations: Similarly to #2, tACE2 shedding by ADAM-17 induced by ANG II is reduced.
4. Reduction or equal levels of ANG II. Origin: Decrease in ANG II levels promoted by drugs, such as ACE inhibitors (30).
5. Mild decrease in ANG 1-7. Origin: Not so pronounced decrease in tACE2 levels (31, 32) concomitantly to a mild increase in biologically active sACE2, combined to a decrease/equal levels of ANG II due to reduced ACE activity.
6. Normal AT1R activation. Origin: Decrease or maintenance in ANG II levels, and/or AT1R downregulation (33–35) (see # 4).
7. Reduction or equal ADAM-17 activity. Origin: Decrease or maintenance in ANG II levels, resulting in equal or reduced AT1R activation (see # 4 and 6).

## Discussion

**Figure 2:**
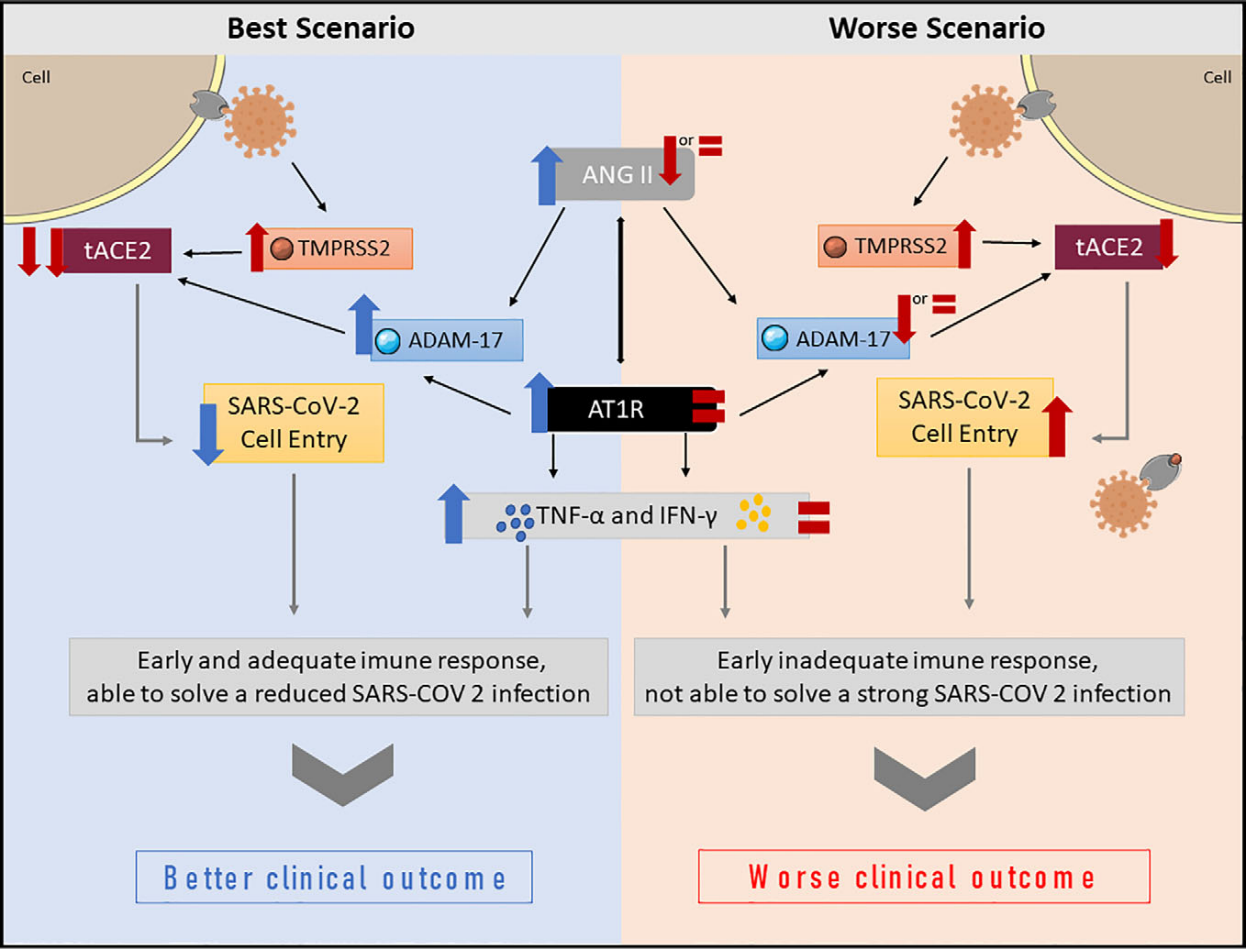
Summary of proposed changes in the RAAS, and downstream effects following SARS-CoV-2 infection. In the best-case scenario, a peak of ANG II occurs immediately after SARS-CoV-2 infects the cell. The virus reduces tACE2 levels due to increased TMPRSS2 and ADAM-17 activity. Reduced tACE2 levels decrease virus entry into the cell, and, consequently, the viral load. The ANG II peak will also increase AT1R activation, modulating not only the ANG II effects, but also the host immune response, which produces TNF-α and IFN-γ in an appropriate time and amount, favoring better clinical outcomes. In the worst-case scenario, the host is unable to elevate ANG II levels. Even with reduced tACE2 due to virus TMPRSS2 activation, ANG II levels remain unaltered or reduced. Consequently, ADAM-17 activation is lower or unaltered, thus, more tACE2 is available, allowing more virus to enter the cell and replicate. Moreover, AT1R activation is also low or unaltered, which leads to an inappropriate early immune response, favoring poor clinical outcomes.

Our hypothesis suggests the RAAS may have a greater role in modulating the physiological response to SARS-CoV-2 infection, and, consequently, to COVID-19 outcomes than previously reported. Here we present two hypothetical scenarios—the best and the worst—involving the RAAS in COVID-19, both of which involve ANG II as a primary response to SARS-CoV-2 infection. All proposed changes are related to the early stage of SARS-CoV-2 infection, being relevant to the onset of the physiological response, and influencing COVID-19 outcomes. **Figure 2** summarizes the proposed hypothesis for both bets and worst-case scenario.

### The Role of the RAAS in the Best-Case Scenario

The best scenario is probably related to a limited entry of the virus into the cell and consequently a better prognosis. The first step implicates TMPRSS2 and ADAM-17 sheddases, which are involved in tACE2 cleavage. The mechanisms leading to tACE2 shedding by TMPRSS2 are different from the ADAM-17 pathway (16). Studies with SARS-CoV suggest tACE2 shedding by the virus involves TMPRSS2 activation in the short cytoplasmic domain of tACE2. By contrast, ADAM-17 constitutively sheds tACE2 to produce its soluble form (sACE2), which retains its biological activity (14, 16, 21).

Regarding SARS-CoV-2 infection, ADAM-17 and TMPRSS2 provoke opposite effects. SARS-CoV-2 has been shown to bind to tACE2, which then is partially or fully internalized to the cell cytoplasm along with the virus (24). In this process, TMPRSS2 is activated, leading to tACE2 shedding and thus reduced tACE2 levels (14, 24, 28, 36, 37). As a consequence, TMPRSS2 activation leads to increased SARS-CoV and SARS-CoV-2 cell penetration in the host cell as well as to increased ANG II levels, as shown in **Figure 1B** (13, 16, 22, 24). It has been shown that African Americans and the elderly present increased levels of TMPRSS2, which might to some extent explain the increased severity of COVID-19 seen in these two populations (19).

Interestingly, ANG II is known to upregulate ADAM-17 activity in different tissues, such as heart muscle, kidney, pancreas, etc (38). In this case, if ANG II and ADAM-17 levels increase, some of the tACE2 may be shedded, which might be an important biological response to reduce SARS-CoV-2 infection in some tissues. Moreover, studies have demonstrated sACE2 generated from ADAM-17 cleavage is capable of binding SARS-CoV-2, sequestrating the virus and preventing it from attaching to the tACE2 (28). Curiously, people from blood group A are at higher risk of developing severe forms of COVID-19 and exhibit lower plasmatic ACE levels (39). Hence, patients with blood group A are more likely to have lower levels of ANG II, negatively regulating AT1R activation and ADAM-17, which seems to support our theory.

In the same context, AT1R activation by ANG II downregulates tACE2 expression, reducing ANG 1-7 and favoring ANG II vasoconstrictor activities (40). Chronic AT1R blockade has been shown to lead to a fivefold increase in tACE2 levels in rat aorta (40). These data indicate tACE2 reduction following AT1R activation by ANG II may trigger a positive feedback mechanism in ANG II regulation (40). Positive feedbacks are self-limiting and are rarely the best biological choice to control essential physiological mechanisms, such as blood pressure control. Nevertheless, an ANG II peak may be an interesting biological strategy to reduce viral entry into the cell *via* tACE2, reducing its expression and attenuating the possible viral damage (**Figure 1B**).

### The Role of the RAAS in the Worst-Case Scenario

The worst-case scenario would occur if ANG II production and/or AT1R activity are reduced for any reason, e.g. using ACE inhibitors (30) (**Figure 1C**). In this case, ADAM-17 would not be consistently activated, leading to two concomitant situations: a) there would be more tACE2 available to facilitate the entry of the virus into host cells, and b) less biologically active sACE2 might result in more circulating free virus, with the potential to enter host cells *via* tACE2. Therefore, the TMPRSS2/ADAM-17 balance would be shifted towards TMPRSS2 activation, thus favoring infection by the virus. Corroborating this idea, recent studies have suggested ACE inhibitors are associated to poor prognosis in COVID-19 patients (41, 42). Paradoxically, a literature review from Mackey et al. (43) concludes direct ANG II suppression with ACE inhibitors would stabilize cell membrane complexes between tACE2 and AT1R, thus reducing the ability of the virus to enter host cells, which would be beneficial in COVID-19. This observation requires additional research and the assessment of intervening variables in infected patients (43).

While increased ANG II levels in COVID-19 has been reported (44), as yet there are no studies addressing sACE2 levels in the disease. A similar increase in ANG II levels is also seen in H5N1 avian influenza and H7N9 patients, indicating this biological response could be common in some viral infections (45, 46). Indeed, tACE2 levels are significantly reduced after H1N1 infection, which is probably related to tACE2 degradation *via* the proteasome pathway (directly cleaved by the influenza neuraminidase protein) (47). Moreover, mice infected with H5N1 show tACE2 expression downregulation in the lung and increased ANG II plasmatic levels, as proposed in our model (45, 46, 48). The literature suggests different viruses, not only SARS-CoV and SARS-CoV-2, might use tACE2 as a carrier. One accepted theory is that bat coronavirus can infect small mammals, such as the palm civet (49). This would suggest this biological defense against viral entry is a well conserved mechanism in mammals. These animals act as intermediate hosts, providing a suitable environment for the coronavirus Receptor Binding Domain (RBD) to develop point mutations compatible with tACE2 binding (50, 51). Studies based on sequences from coronavirus found in bats suggest their RBDs contain deletions spanning key residues needed to mediate viral and tACE2 interaction (49, 52).

Several studies corroborate the occurrence of the tACE2 and sACE2 changes proposed in **Figure 1B** (14, 24, 36, 37). The increased sACE2 resultant from TMPRSS2 cleavage, and the decrease in its biologically active form from ADAM-17 cleavage, will lead to a mild increase in sACE2, which might not be sufficient to generate ANG 1-7 from the already low levels of ANG II, which is proposed in **Figure 1C**. Moreover, sACE2 from TMPRSS2 will favor increased SARS-CoV-2 infection in the host cell (21).

These physiological changes produce an imbalance favoring the ACE/Ang II/AT1R axis, which is a pro-inflammatory, vasoconstrictor, fibrotic pathway, while reducing the ACE2/ANG 1-7/MasR signaling pathway that promotes vasodilatation and anti-inflammatory effects (53). Indeed, a recent study suggests the activation of the ACE2/ANG 1-7/MasR axis in order to prevent excessive inflammatory response, especially in lungs (54). Currently, there are three ongoing clinical trials to evaluate the effects of ANG 1-7 administration on hyper-inflammatory response in critically ill COVID-19 patients (55).

sACE2 is a marker of severity and clinical prognosis in several inflammatory diseases. In patients with chronic obstructive coronary artery disease, sACE2 levels were higher than in healthy controls, which was considered an independent predictor of cardiovascular mortality (53). sACE2 levels have been shown to increase in type II diabetes mellitus and coronary arterial disease, being a potential marker for atherosclerosis (56). Also, increased sACE2 levels are considered a marker of poor cardiac outcomes in several cardiovascular diseases (57). Pro-inflammatory cytokines IL1-B and TNF- α also induce ADAM-17 activation, thus increasing sACE2 levels (16). It is worth noting that patients with obesity, diabetes and cardiovascular diseases are risk groups for COVID-19, presenting the worst outcomes (58, 59). Some interesting hypotheses may be related to poor prognosis. First, in patients with diabetes and cardiovascular diseases, ANG II plasmatic levels are typically high, indicating the RAAS is hyperactive (60). Consequently, it would be unlikely to produce a new peak of ANG II to combat the virus infection. Second, the increased ANG II plasmatic levels may also lead to AT1R downregulation. These two hypotheses support the patterns of ANG II and AT1R levels proposed in **Figure 1C**.

An observation should be made regarding the circadian variations in the levels of the RAAS components, which might also affect COVID-19 outcomes (61). It has been shown, in both animals and humans, that several components of the RAAS present a circadian pattern throughout the day (62–64); in mice, for example, both ACE and tACE2 expression follows a circadian pattern, and the ACE/tACE2 ratio is reduced during the night (65). The pathogenicity of a virus has also been shown to be affected by the circadian rhythm through direct regulation of viral replication inside the host cell (61). It is not only the time of day in which the infection occurs that influences the disease outcome, but also disruption of the circadian rhythm increases the viral replication, as observed in herpes and influenza A infections (66). This suggests that SARS-CoV-2 pathogenicity might be influenced by time of infection due to circadian alterations in the ACE/tACE2 ratio. A lower ACE/tACE2 ratio means reduced ANGII levels and increased tACE2, lending SARS-CoV-2 increased pathogenicity; while with a high ratio, the virus would be less pathogenic. This is also supports our hypothesis that ANG II peaks in response to SARS-CoV-2 infection. Since ACE2 counteracts the ANG II effects by cleaving it to ANG 1-7, a high ACE/tACE2 ratio would favor a peak in ANG II levels. In fact, a study by Liu and colleagues (2020), observed markedly high ANG II levels in COVID-19 patients when compared to healthy controls. Moreover, these levels were negatively correlated to both viral load and lung injury, suggesting an important role for ANG II during early SARS-CoV-2 infection (44).

### The Role of the RAAS in the Immune Response in COVID-19

#### The Role of ANG II in Neutrophilic Inflammation

The massive neutrophilic infiltration observed in the lungs from critically ill patients has an important role as an early response to infections (67). In this sense, ANG II plays a role in attracting neutrophils to the lung, since it recruits pro-inflammatory cells to the site of infection (68). Not only is ANG II a chemotaxis factor for mononuclear cells, it also upregulates both chemoattractant cytokines and adhesion molecules such as P-selectin, Intercellular cell adhesion molecule type I (ICAM I) and Vascular cell adhesion molecule type I (VCAM), on vascular endothelial cells and smooth muscle cells, leading monocytes and neutrophils to adhere to endothelial cells (69, 70). An increase in ANG II is one of the early responses during inflammatory processes (68). However, these processes are counterbalanced by the sACE2 levels, which are pivotal in controlling neutrophilic infiltration in lungs (71). During the onset of infection, the combination of reduced sACE2 and increased ANG II trigger neutrophilic infiltration to the lungs and initiation of inflammatory processes. The ANG II peak increases ADAM-17 *via* AT1R activation, leading to increased sACE2 and favoring the ANG 1-7/MasR pathway, modulating the ongoing inflammation and preventing further tissue damage (71–73). Taken together, we can speculate that, in COVID-19, the inflammatory process, in lungs, is also importantly coordinated by both ANG II and sACE2 levels. In best-case scenario with COVID-19, since SARS-CoV-2 uses tACE2 to enter the cell, its levels are reduced and ANG II peaks as an early response to SARS-CoV-2 infection, triggering an inflammatory response to fight the viral infection. In the lungs, sACE2 levels will start to increase in response to viral entry in the cell. The sACE2, produced by ADAM-17, will both bind to viral particles blocking their entry into the cells, and thus protecting the organism from infection, while also cleaving ANG II, thus controlling both the neutrophilic infiltration and the inflammatory response. After which, late immune responses take place in order to control the infection. By contrast, in the worst-case scenario, even with reduced tACE2 levels, the individual is unable to produce an ANG II peak in response to early infection, with the levels remaining normal or even reduced. Thus, the early neutrophilic response necessary to fight the viral infection does not take place, or is delayed. In a mouse model of bacterial lung infection, it has been shown that reduced neutrophilic infiltration will not trigger the inflammatory response necessary for infection resolution (71). Once infection takes place in the lungs, sACE2 levels start to rise, cleaving ANG II and reducing its levels even more, resulting in a delayed, or even blunted, immune response to early infection concomitantly to the viral proliferation.

#### ANG II and T Lymphocyte Activation

**Figure 3:**
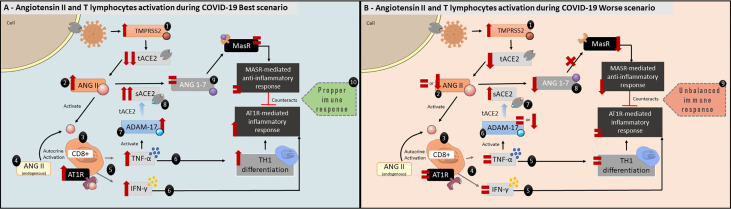
Angiotensin II and T lymphocytes activation during COVID-19 best scenario. **(A)** 1: Reduction in tACE2 following SARS-CoV-2 infection. 2: ANG II will increase and 3: act as a co-stimulatory molecule in T lymphocytes activation acting *via* AT1R, which are expressed in CD8+ cells. 4: ANG II is also expressed by T lymphocytes, enhancing AT1R activation in an autocrine and intracrine manner. 5:AT1R activation stimulates CD8+ cells to produce both TNF-α and IFN-γ. 6: TNF-α will increase TH1 differentiation, and, together with IFN-γ, activate the inflammatory response. 7: TNF- α will also activate ADAM-17 activity leading to 8: increase the biologically active sACE2 and, 9: maintaining ANG 1-7 levels. 10: ANG 1-7, which has anti-inflammatory characteristics, will counterbalance TNF-α and IFN-γ direct effects *via* MasR pathway. Taken together, these alterations would lead to a proper immune response to SARS-CoV-2 infection. **(B)** Angiotensin II and T lymphocytes activation during COVID-19 worse scenario. 1: tACE2 levels are reduced following SARS-CoV-2 infection. 2: ANG II levels remain the same or are even low (see **Figure 1C**, COVID-19 Worse Scenario), 3: maintaining the AT1R activation in CD8+ cells at normal levels. 4: As a result, IFN-γ and TNF-α levels remain the same, 5: affecting the beginning of inflammatory response. 6: ADAM-17 activity is equal or reduced, 7: leading to reduced biologically active sACE2 levels. 8: consequently, ANG 1-7 levels are reduced. 9: This unbalanced immune response could not be adequate to combat SARS-CoV-2 infection.

ANG II also plays a role in inflammatory responses, acting as a co-stimulatory molecule to T lymphocyte activation, promoting cellular proliferation, differentiation, effector function, migration and adhesion (74–76). The T lymphocytes are able to produce both AT1R and ANG II, activating immune cells *via* autocrine mechanisms (**Figure 3**) (75, 76). Moreover, *in vitro* and *in vivo* experiments demonstrated that AT1R expression in T lymphocytes is upregulated during the activation process. This finding suggests ANG II plays a role in augmenting immune responses (75–77). AT1R is expressed in both CD4+ and CD8+ cells, which are T helper lymphocytes and T cytotoxic cells, respectively (76). Attention should be paid to AT1R expression in CD8+ T cells, which are effectors in antiviral responses (78). Interestingly, AT1R is also expressed inside T cells, which suggests that endogenously produced ANG II may act in those receptors *via* the intracrine pathway (75).

ANG II autocrine and intracrine AT1R activation will stimulate superoxide production which, in turn, stimulates TNF-α production (**Figure 3**) (75). In addition, TNF-α upregulates AT1R, thus enhancing ANG II signaling (79). Even though TNF-α is produced by several immune cells such as macrophages, B cells and neutrophils, TNF-α production by T cells has an important role during antiviral adaptive responses, since it promotes apoptosis of infected cells as well as modulating the CD8+ T cells effector phase during viral infections (80, 81). AT1R and AT2R blockade or deficiency show significantly reduced production of TNF-α by T cells in mice models (75). Moreover, ANG II induces T cells to express interferon-γ (IFN-γ), one of the first cytokines to be produced in antiviral responses, thus inducing immune cell differentiation to a TH1 phenotype (i.e., an inflammatory profile—**Figure 3A**) (76, 82). Interestingly, a delayed IFN-γ response leads to exacerbated inflammation in mice infected with SARS-CoV and MERS (83, 84). This also supports our hypothesis, as lower ANG II levels would lead to unaltered or lowered IFN-γ production by CD8+ cells (**Figure 3B**).

### Immune Exhaustion

Regarding T lymphocytes, a marked decrease in CD3+, CD4+ and CD8+ T cells have been shown in critically ill patients (28, 85, 86). The CD8+ decrease may be significant in patients with death outcome in comparison with those who recovered. Recent studies (85) demonstrated that cytotoxic CD8+ T cells from COVID-19 patients presented severe inflammatory and antiviral activity compared to healthy controls (85, 87, 88). Therefore, reduction in CD8+ T cells observed in COVID-19 might actually represents an exhaustion-related effect in this cell subpopulation due to clonal expansion of activated cells. As a result, there is an imbalance between the adaptive response and the disease progression.

The RAAS has a critical role in the early response to SARS-CoV-2 infection and, consequently, COVID-19 better or worse prognoses. In addition, ANG II plays an important role in the onset and progression of inflammatory responses. Taken together, it is possible that an ANG II peak is our first biological response against virus entry (89, 90). To the best of our knowledge, this is the first viewpoint to explore the logical interaction of these pathophysiological mechanisms. Thus, monitoring ACE, tACE2, sACE2, ANG I, ANG II, ANG 1-7, and ANG 1-9 during COVID-19 may provide clues to develop effective treatments.

## Data Availability Statement

The original contributions presented in the study are included in the article/supplementary material. Further inquiries can be directed to the corresponding author.

## Author Contributions

LX, PN, and AW—study design. data collection, and interpretation, manuscript elaboration and review. LP, LN, PB, LT, AR-F, and RM—data collection and interpretation. LX, PN, LP, and AW—figures conception and elaboration. All authors contributed to the article and approved the submitted version.

## Funding

This study was financed in part by Coordenação de Aperfeiçoamento de Pessoal de Nível Superior-Brasil (CAPES)—Finance Code 001; Conselho Nacional de Pesquisa e Desenvolvimento (CNPq)—Grant Numbers 306644/2016-9 and 423884/2018-2; Fundação de Amparo à Pesquisa do Estado do Rio Grande do Sul (FAPERGS). LX and AR-F are CNPq researchers.

## Conflict of Interest

The authors declare that the research was conducted in the absence of any commercial or financial relationships that could be construed as a potential conflict of interest.
